# Longitudinal Brain Changes After Stroke and the Association With Cognitive Decline

**DOI:** 10.3389/fneur.2022.856919

**Published:** 2022-06-03

**Authors:** Eva B. Aamodt, Stian Lydersen, Dag Alnæs, Till Schellhorn, Ingvild Saltvedt, Mona K. Beyer, Asta Håberg

**Affiliations:** ^1^Institute of Clinical Medicine, University of Oslo, Oslo, Norway; ^2^Division of Radiology and Nuclear Medicine, Oslo University Hospital, Oslo, Norway; ^3^Regional Centre for Child and Youth Mental Health and Child Welfare, Department of Mental Health, NTNU – Norwegian University of Science and Technology, Trondheim, Norway; ^4^Norwegian Centre for Mental Disorders Research (NORMENT), Division of Mental Health and Addiction, Oslo University Hospital and Institute of Clinical Medicine, University of Oslo, Oslo, Norway; ^5^Department of Neuromedicine and Movement Science, Faculty of Medicine and Health Science, NTNU – Norwegian University of Science and Technology, Trondheim, Norway; ^6^Department of Geriatrics, Clinic of Medicine, St. Olavs Hospital, Trondheim University Hospital, Trondheim, Norway; ^7^Department of Radiology and Nuclear Medicine, St. Olavs Hospital, Trondheim University Hospital, Trondheim, Norway

**Keywords:** morphology, atrophy, post-stroke dementia, neuroimaging, neurocognitive disorder

## Abstract

**Background:**

Cognitive impairment is common after stroke. So is cortical- and subcortical atrophy, with studies reporting more atrophy in the ipsilesional hemisphere than the contralesional hemisphere. The current study aimed to investigate the longitudinal associations between (I) lateralization of brain atrophy and stroke hemisphere, and (II) cognitive impairment and brain atrophy after stroke. We expected to find that (I) cortical thickness and hippocampal-, thalamic-, and caudate nucleus volumes declined more in the ipsilesional than the contralesional hemisphere up to 36 months after stroke. Furthermore, we predicted that (II) cognitive decline was associated with greater stroke volumes, and with greater cortical thickness and subcortical structural volume atrophy across the 36 months.

**Methods:**

Stroke survivors from five Norwegian hospitals were included from the multisite-prospective “Norwegian Cognitive Impairment After Stroke” (Nor-COAST) study. Analyses were run with clinical, neuropsychological and structural magnetic resonance imaging (MRI) data from baseline, 18- and 36 months. Cortical thicknesses and subcortical volumes were obtained via FreeSurfer segmentations and stroke lesion volumes were semi-automatically derived using ITK-SNAP. Cognition was measured using MoCA.

**Results:**

Findings from 244 stroke survivors [age = 72.2 (11.3) years, women = 55.7%, stroke severity NIHSS = 4.9 (5.0)] were included at baseline. Of these, 145 (59.4%) had an MRI scan at 18 months and 72 (49.7% of 18 months) at 36 months. Most cortices and subcortices showed a higher ipsi- compared to contralesional atrophy rate, with the effect being more prominent in the right hemisphere. Next, greater degrees of atrophy particularly in the medial temporal lobe after left-sided strokes and larger stroke lesion volumes after right-sided strokes were associated with cognitive decline over time.

**Conclusion:**

Atrophy in the ipsilesional hemisphere was greater than in the contralesional hemisphere over time. This effect was found to be more prominent in the right hemisphere, pointing to a possible higher resilience to stroke of the left hemisphere. Lastly, greater atrophy of the cortex and subcortex, as well as larger stroke volume, were associated with worse cognition over time and should be included in risk assessments of cognitive decline after stroke.

## Introduction

Although the chances of surviving a stroke have become high, its aftermath often involves the development of cognitive problems (1). About 30% of stroke survivors develop cognitive impairments after a first-ever stroke, with the incidence of dementia within the first year after a major stroke being almost 50 times higher than in the general population, and nearly 6 times higher after a minor one (1). Stroke and dementia both pose risks for each other and share many of the same neurodegenerative and cerebrovascular risk factors (2), with pre-existing structural brain pathology being a frequent finding in stroke patients (3). Early onset (<3 months) of post-stroke dementia (PSD) has been found to be primarily associated with stroke lesion volume and white matter hyperintensity (WMH) load (4–7). The likelihood of developing cognitive impairment after a stroke does however remain elevated for many years after the incident (8), with late onset (>6 months) being mainly associated with the presence of lacunes and a history of ischemic heart disease and stroke (9). Moreover, early deficits in cognition after a stroke does not necessarily mean long-term problems and some studies report that about ¼ of patients with cognitive impairment at time of discharge show improvement already within the first year after a stroke event (10). Discovering reliable methods of identifying stroke patients at risk of dementia vs. only transitory cognitive impairment therefore has major implications in the development of tailorized follow-up strategies for patients, thereby decreasing both the personal and the societal disease burden of PSD.

Accelerated post-stroke cortical and subcortical atrophy is common, but the pattern it occurs in remains somewhat unclear. Stroke has been found to be associated with an overall accelerated atrophy rate for at least 3–12 months after a stroke incident, with the atrophy taking place irrespective of stroke lesion location (11). This could either be explained by distant neurophysiological changes, also called diachisis (12) or by secondary atrophy, also called Wallerian degeneration, affecting descending fiber tracts from the locus of the stroke, leading to structural changes also in areas distal to the stroke (13). In stroke there may also be a greater atrophy rate in the ipsi- (same side as the stroke) than the contralesional (opposite side of the stroke) hemisphere. Ipsilesional cortices and subcortical structures have been found to decrease more than its contralesional counterparts (11, 14), although some structures, such as the hippocampus, have also been reported to shrink equally in both hemispheres (15). Additionally, some studies report that the ipsilesional hemisphere atrophies more than the contralesional hemisphere only if the stroke was located to the middle cerebral artery territory (16). These somewhat diverging findings call for further clarification.

Although the exact spatial pattern of atrophy remains unclear, the effects of a stroke has been shown to last for years, with findings showing persistent greater brain atrophy up to 6 years after a stroke insult (17). Moreover, ipsilesional cortical and subcortical atrophy as opposed to contralesional atrophy rates have been found to be 2–4 times greater at 1 year compared to at 3 months after a stroke (11). These findings suggest that a stroke leads to protracted atrophy in the ipsilesional cortex more so than in other parts of the brain. Still, the temporal profile of the atrophy is yet to be fully understood. Longitudinal designs are therefore required to map out a potential difference in atrophy pattern trajectories between anatomical regions following a stroke.

Both cortical and subcortical atrophy have been shown to be associated with cognitive decline (14, 18). The brain changes reported as providing the greatest risk of cognitive decline in cross-sectional and longitudinal studies lasting up to 3–12 months are global brain atrophy, medial temporal lobe (including hippocampal) atrophy, and WMH load (4, 19–22). Since accelerated brain atrophy is demonstrated for a longer time period, potentially important changes in brain structure-function relationship over time may be overlooked due to relatively short follow-up times in previous studies. The current study therefore aimed to use a longitudinal follow-up design including both cognition and structural brain imaging assessed three times across 36 months in contrast to the typical 3–12 months follow-up.

The current prospective 36-months follow-up study of stroke patients aimed to investigate the longitudinal associations between (I) brain structure atrophy and stroke hemisphere, and (II) cognitive impairment and brain structure atrophy after stroke. We expected to find that (I) cortical thickness and hippocampal-, thalamic-, and caudate nucleus volumes declined more in the ipsilesional than the contralesional hemisphere at both 18- and 36 months after stroke. We also expected that (II) cognitive decline was associated with a larger stroke volume, and with greater cortical thickness and hippocampal-, thalamic-, and caudate nucleus volume atrophy at each timepoint.

## Materials and Methods

### Nor-COAST

The current study is based on data from the Norwegian Cognitive Impairment After Stroke study (Nor-COAST)—a prospective longitudinal multicenter cohort study recruiting patients hospitalized with acute stroke at five Norwegian stroke units (23). Patient recruitment started in May of 2015 and was completed in March of 2017. Details of the Nor-COAST study are described elsewhere (23). The study was approved by the regional committee for medical and health research, REK Nord (REK Number: 2015/171), and registered on clinicaltrials.gov (NCT02650531). REK Nord has also approved this current sub study (REK Number: 2019/397). All participants provided written informed consent in accordance with the Declaration of Helsinki. If a potential participant was unable to give consent, written informed consent for participation was given by a family proxy.

### Subjects

Inclusion criteria for Nor-COAST were (a) patients admitted with acute ischemic or hemorrhagic stroke hospitalized within 1 week after onset of symptoms, diagnosed according to the World Health Organization (WHO) criteria; (b) age over 18 years; (c) fluent in a Scandinavian language.

Exclusion criteria for Nor-COAST were (a) not treated in the participating stroke units; (b) symptoms explained by other disorders than ischemic brain infarcts or intracerebral hemorrhages; (c) expected survival <3 months after stroke.

Additional inclusion criteria for MRI sub study were (a) modified Rankin scale <5 before the stroke; (b) able to cooperate during MRI.

Exclusion criteria for MRI sub study were (a) severe functional impairment making MRI impossible to perform; (b) medical contraindications for MRI like claustrophobia or pacemaker; (c) patient declining participation in MRI.

### MRI Acquisition

A study-specific brain MRI was performed as soon as an MRI-scanner was available during the acute/subacute phase of the stroke (i.e., within 2–7 days), as well as during follow-up at 18- and 36 months. Brain scans were acquired at five different hospitals, using a single MRI-scanner at each site (GE Discovery MR750, 3T; Siemens Biograph_mMR, 3T; Philips Achieva dStream, 1.5T; Philips Achieva, 1.5T; Siemens Prisma, 3T). The study protocol consisted of 3D-T1 weighted, axial T2, 3D-Fluid attenuated inversion recovery (FLAIR), diffusion-weighted imaging (DWI), and susceptibility-weighted imaging (SWI). Detailed description of the MRI protocol can be found in Supplementary Table 1. Causes of why patients declined participation in the MRI sub study were not recorded, in accordance with the ethical approval.

### Data Preparation

Cortical thickness and subcortical (including the allocortical structure hippocampus) volumetric measurements were generated through cortical reconstruction and parcellation, and volumetric segmentation of the 3D-T1 scans. This was performed using the comprehensive longitudinal recon-all process of Freesurfer 6.0.1 image analysis suite (http://surfer.nmr.mgh.harvard.edu/) (24). Cortical measurements were gathered into lobes (frontal, medial temporal, lateral temporal, and parietal) as suggested by Klein and Tourville (25), and hemispheres (by adding cortex, subcortex and white matter). Quality control of the FreeSurfer output was conducted based on the ENIGMA protocol [http://enigma.ini.usc.edu; (26)] and cross-validated using Euler scores derived from the longitudinal FreeSurfer process. The QC revealed relatively good results with exclusion of 63 of the total 704 scans (8.95%) across all timepoints with 37 excluded due to Freesurfer error and 26 due to failing QC.

### Stroke Location, Stroke Volume and WMH Volume Extraction

Stroke location was based on the lesion masks and determined using the Talairach lobe atlas (27, 28). The labels “anterior lobe” and “posterior lobe” were then gathered into “Cerebellum”, and “medulla”, “midbrain”, and “pons” were gathered into “Brainstem”. If the stroke location was labeled as “background”, the Harvard-Oxford structural atlas (29–32) was used instead. Stroke lobe location was determined by the lobe with the highest percentage of probability. The same principle was used for cases with multiple unilateral lesions. Participants with bilateral stroke was excluded from analysis I.

A selection of cortices and subcortical structures to be used in the analyses was made based on previous literature (14, 18–20, 22).

Stroke volume lesion masks were created from the DWI scans at baseline for the patients who had visible diffusion restriction on DWI. The DWI lesion volume is equivalent to the ischemic core that is a proxy of the amount of irreversibly destroyed brain parenchyma, identified as diffusion restriction on the DWI sequence. The acute infarcts were semi-automatically segmented with the help of the ITK-Snap snake tool (v. 3.8.0) (33). The masked stroke volume in mm^3^ was automatically calculated by ITK-snap and converted to mL. Cases with no positive ischemic DWI lesions were not included in the study.

WMH segmentation was performed using the fully automated and supervised FMRIB tool FSL BIANCA (34). Due to noticeable underestimation of WMH across multiple thresholds, manual editing of the FSL BIANCA output was performed. Full description of WMH segmentation methods is given elsewhere (5).

### Clinical Characteristics

Demographic- and clinical data were collected by study nurses and stroke physicians at the time of the index stroke, as well as at the 18- and 36 months follow-up. Based on previous literature on factors associated with neurodegeneration and post-stroke cognitive impairment, the following factors were included in the analyses; age, education (years), sex, waist-to-hip ratio (WHR), stroke severity (at admission) measured using the “National Institute of Health Stroke Scale (NIHSS), atrial fibrillation (AF) and diabetes status at baseline, and pre-stroke hypertension and –global cognition, as measured through the global deterioration scale (GDS). Cognitive outcome was assessed using the education-adjusted Montreal Cognitive Assessment (MoCA) scale (35). Disability was measured using the modified Rankin scale (mRs) and comorbidity was measured using the Charlson comorbidity index (CCI). A high WHR was characterized as a ratio of >0.86 in women and >1.00 in men (36). The NIHSS scale ranges from 0 to 42, with higher scores indicating more severe strokes. AF was defined as patients having a (past or present) pathological ECG recording. The GDS scale (37) ranges from 1 to 7 and was collected through interviews with relatives or caregivers. The MoCA scale ranges from 0 to 30, with higher scores indicative of better cognition (35).

### Statistical Analysis

Descriptive statistics are reported as mean and standard deviations (SD) or n (%). We compared stroke lesion volume, age, disability (mRs), comorbidity (CCI), sex, diabetes mellitus, hypertension and smoking status between individuals with left vs. right stroke hemisphere using descriptive statistics and *t*-tests or Pearson chi squared tests.

Two sets of analyses were performed;

- *The association between ipsilesional vs. contralesional stroke hemisphere and longitudinal cortical and subcortical atrophy*:
  Linear mixed model analyses were performed per cortical thickness/subcortical volume, per hemisphere, one at a time, as dependent variables (e.g., left hippocampus). The models included the following covariates: ipsilesional vs. contralesional hemisphere, time-point, and their interactions. The analyses were adjusted for the possible confounders: age, sex, scanner, and estimated total intracranial volume (etiv; if the dependent variable was volumetric).
  In addition, we performed the analyses mentioned above now including both left and right hemisphere in the same model (e.g., hippocampus), adding right vs. left hemisphere and its interaction with ipsilesional vs. contralesional hemisphere, time-point, and their three-way interactions, as covariates.
- *The association between longitudinal cortical and subcortical atrophy/stroke size and cognitive impairment*:
  Linear mixed model analyses were performed with cognition as the dependent variable and with cortical thickness/subcortical volume or stroke volume, one at a time (e.g., hippocampus), as independent variable. The models also included the following covariates; stroke hemisphere, time-point, and their interactions. These analyses were adjusted for the possible confounders: age, sex, scanner, and education.
  In addition, we performed the analyses mentioned above now with the added possible confounder of WMH volume.

The proportion missing data at different time points depended on age and other observed covariates. Values are therefore not missing completely at random (MCAR), but are possibly missing at random (MAR). The use of linear mixed models provides unbiased estimates under the less restrictive MAR assumption.

Normality of residuals was checked through visual inspection of QQ-plots. Bootstrapping with B = 1,000 bootstrap samples and the bias-corrected and accelerated (BCa) intervals were done for eight models (thalamus and caudate nucleus) that showed non-normal residuals. Due to multiple hypotheses we used a two-sided significance level of 1% to give some protection against false positive findings (38). Analyses were performed in Stata 16.1.

## Results

### Study Population

**Figure 1:**
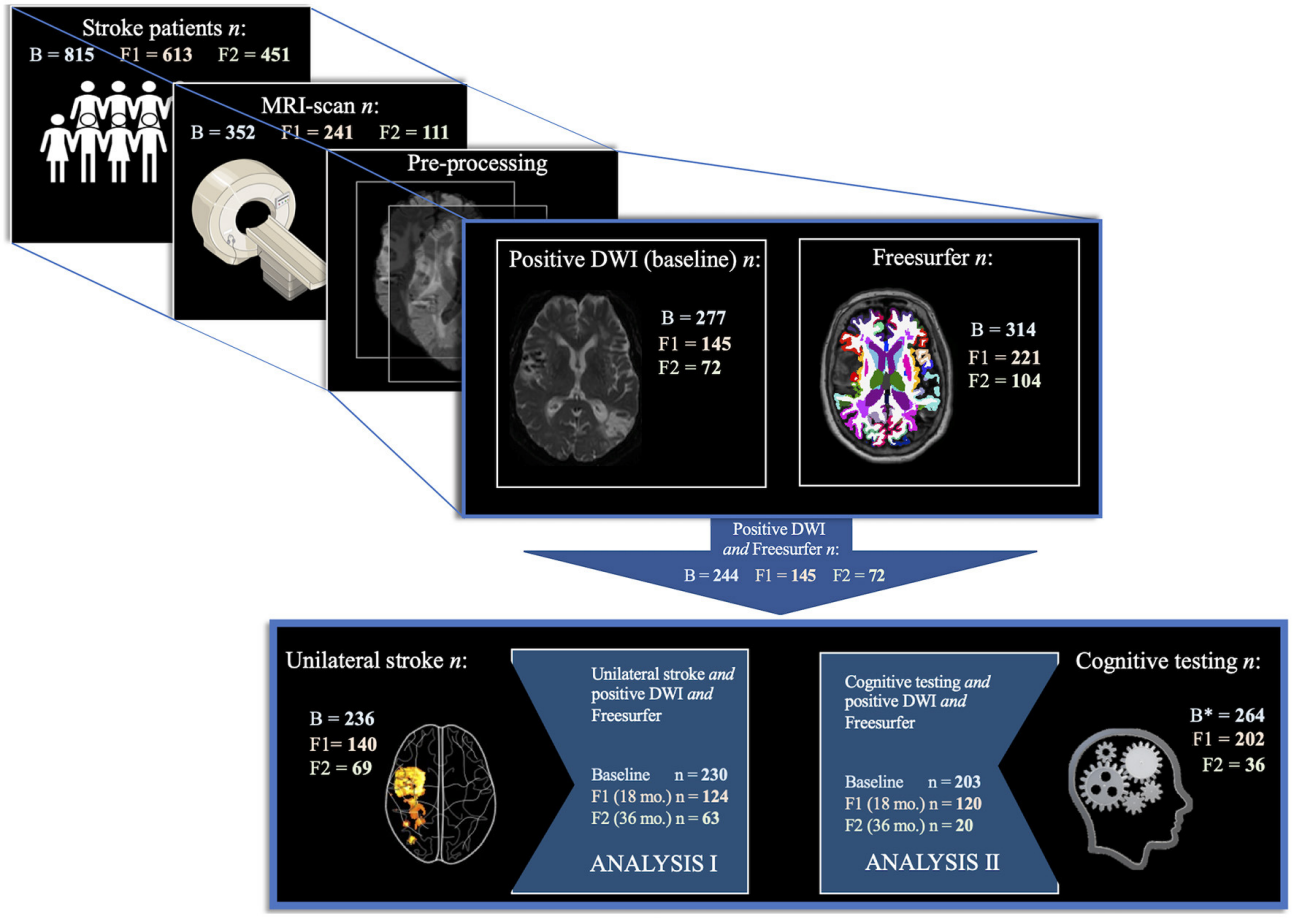
Study population selection. Study population selection by analysis per timepoint. The figure depicts the inclusion process from 815 Nor-COAST participants at baseline, to 352 included in the MRI sub-study, whereupon 277 of these also had the stroke lesion visible on DWI, and 314 had successful Freesurfer anatomical segmentation, resulting in a final study sample of 244 at baseline. Out of these, 230 had a unilateral stroke and was used in analysis I. Out of the final study sample, 203 had cognitive testing at 3 months and was used in analysis II. B, Baseline. B^*^, Baseline (3 months). F1, Follow-up 1 (18 months). F2, Follow-up 2 (36 months).

From the complete Nor-COAST population, 352 (43.2%) underwent a study-specific MRI scan that fulfilled all quality requirements for further analysis. Of the included participants, 157 (44.6%) were women, the age was 72.8 (11.2) years and admission stroke severity NIHSS scores were 4 (4.9). Positive DWI, enabling stroke lesion segmentation, was available in 277 (78.7%) of these, and Freesurfer anatomical segmentation failed only in 38 (10.8%) participants, resulting in a final study sample of 244 (69.3%) at baseline, 145 (59.4% of baseline) at 18 months and 72 (49.7% of 18 months) at 36 months (see Figure 1 for more follow-up numbers). The final study sample at baseline consisted of 136 (55.7%) women, with the age of 72.2 (11.3) years, and stroke severity NIHSS score of 4.9 (5.0).

### Baseline Clinical and Stroke Characteristics

For the final study sample population at baseline, a WHR ratio of 0.89 (.07) was found for the men and 0.98 (0.07) for the women. High sex-specific WHR scores were found in 151 (71.2%) of the participants. Hypertension was present in 120 (49.2%), atrial fibrillation (AF) in 50 (20.5%), and diabetes in 50 (20.5%), of the participants. GDS scores of 1.5 (0.86) (min = 1, max = 5) revealed an average between no to very mild decline of cognition prior to the stroke. Admission stroke severity NIHSS scores generally fell within the “mild stroke” category, with a score of 4.1 (5.0) (min = 0, max = 42). For characteristics of participants included in analysis I and II, see Table 1.

**Table 1:** Participant characteristics, mean (SD) or *n* (%).

	**Analysis I Participants with unilateral stroke**			**Analysis II Participants with cognitive scores**		
**Variable**	**Baseline**	**18 months**	**36 months**	**Baseline**	**18 months**	**36 months**
***N***	**230**	**124**	**63**	**203**	**120**	**20**
**Demographic and clinical characteristics**						
Age (years)	72.3 (11.3)	72.0 (10.7)	74.4 (9.5)	71.0 (11.1)	71.7 (10.2)	76.8 (9.0)
Education (years)	12.1 (3.7)	12.4 (3.9)	12.4 (4.0)	12.4 (3.7)	12.3 (3.8)	12.4 (3.7)
Women	128 (55.6%)	70 (56.5%)	39 (61.9%)	112 (55.2%)	70 (58.3%)	12 (60%)
High WHR^*^	139 (69.5%)	72 (74.2%)	15 (79.0%)	124 (69.3%)	77 (75.5%)	15 (83.3%)
NIHSS (T0)^*^	3.9 (4.7)	4.0 (5.2)	4.0 (6.1)	3.6 (4.5)	3.7 (4.9)	3.2 (3.4)
Atrial fibrillation (at baseline)	46 (20.0%)	25 (20.2%)	10 (15.9%)	39 (19.2%)	24 (20%)	3 (15%)
Diabetes (at baseline)	43 (18.7%)	20 (16.1%)	6 (9.5%)	39 (19.2%)	21 (17.5%)	1 (5%)
Hypertension (pre-stroke)	112 (48.7%)	56 (45.2%)	28 (44.4%)	97 (47.8%)	56 (46.7%)	9 (45%)
GDS (pre-stroke)	1.5 (0.9)	1.3 (0.6)	1.2 (0.4)	1.4 (0.7)	1.3 (0.6)	1.2 (0.4)
MoCA	24.6 (4.3) ^*^	25.3 (3.7) ^*^	25.3 (4.0) ^*^	24.6 (4.3)	25.2 (3.8)	25.3 (4.0)

*Demographic and clinical findings for baseline, 18 months and 36 months, both for participants included in analysis I and participants included in analysis II. WHR, Waist-hip ratio; High WHR, >0.86 in women and >1.00 in men; NIHSS, National Institutes of Health Stroke Scale; GDS, Global Deterioration Scale; MoCA, Montreal Cognitive Assessment*.

* *Missing data*.

Average stroke lesion volume from DWI scans was 12.2 (29.1) mL. The most frequent stroke locations were the frontal cortex (29.2%) and subcortical structures (20.4%). No significant difference in proportion between (unilateral) stroke hemisphere was found, with 49.2% right-sided strokes and 47.5% left-sided strokes. Only 8 (3.3%) participants had acute strokes in both hemispheres and were excluded from analysis I. For MRI findings of participants included in analysis I and II, see Table 2. For stroke characteristics, see Supplementary Table 2.

**Table 2:** Participant's structural MRI measurements, mean (SD).

	**Analysis I Participants with unilateral stroke**			**Analysis II Participants with cognitive scores**		
**Variable *N***	**Baseline 230**	**18 months 124**	**36 months 63**	**Baseline 203**	**18 months 120**	**36 months 20**
**Quantitative MRI results**						
eTIV (mL)	1,503.6 (166.0)	1,507.6 (159.1)	1,514.9 (159.8)	1,511.3 (166.6)	1,508.5 (157.7)	1,519.7 (188.4)
Left brain volume—ipsilateral stroke (mL)	455.40	-	-	-	-	-
Left brain volume—contralateral stroke (mL)	449.87					
Right brain volume—ipsilateral stroke (mL)	444.68	-	-	-	-	-
Right brain volume—contralateral stroke (mL)	456.47					
Ipsilateral brain volume (mL)	450.04					
Contralateral brain volume (mL)	453.17					
Stroke lesion (mL) (baseline)	12.2 (29.5)	10.4 (28.3)	7.6 (17.5)	8.6 (17.6)	8.2 (16.2)	8.8 (15.0)
Frontal th. (mm)	-	-	-	2.39 (0.11)	2.38 (0.12)	2.40 (0.12)
Frontal th. (left) (mm)	2.38 (0.12)	**2.39 (0.12)**	**2.40 (0.11)**	-	-	-
Frontal th. (right) (mm)	2.38 (0.12)	2.37 (0.12)	2.38 (0.12)	-	-	-
Med. Temp. th. (mm)	-	-	-	2.88 (0.17)	2.90 (0.16)	2.89 (0.19)
Med. Temp. th. (left) (mm)	2.85 (0.20)	2.88 (0.22)	2.88 (0.18)	-	-	-
Med. Temp. th. (right) (mm)	**2.87 (0.19)**	**2.90 (0.18)**	**2.91 (0.18)**	-	-	-
Lat. Temp. th. (left) (mm)	2.45 (0.15)	2.46 (0.13)	2.44 (0.13)	-	-	-
Lat. Temp. th. (right) (mm)	**2.47 (0.13)**	**2.49 (0.13)**	**2.47 (0.14)**	-	-	-
Parietal th. (left) (mm)	2.21 (0.12)	2.22 (0.11)	**2.20 (0.11)**	-	-	-
Parietal th. (right) (mm)	2.21 (0.12)	2.22 (0.12)	2.19 (0.11)	-	-	-
Hippocampal vol. (mL)	-	-	-	3.60 (0.58)	3.60 (0.55)	3.56 (0.58)
Hippocampal vol. (left) (mL)	3.52 (0.61)	3.53 (0.62)	3.56 (0.56)	-	-	-
Hippocampal vol. (right) (mL)	**3.60 (0.60)**	**3.62 (0.60)**	**3.62 (0.53)**	-	-	-
Thalamic vol. (mL)	-	-	-	6.50 (0.90)	6.27 (0.83)	6.10 (0.85)
Thalamic vol. (left) (mL)	**6.46 (1.00)**	**6.29 (0.97)**	**6.25 (0.82)**	-	-	-
Thalamic vol. (right) (mL)	6.37 (0.86)	6.20 (0.94)	6.10 (0.85)	-	-	-
Caudate vol. (mL)	-	-	-	3.78 (0.75)	3.60 (0.63)	3.52 (0.81)
Caudate vol. (left) (mL)	3.70 (0.79)	3.53 (0.69)	3.56 (0.69)	-	-	-
Caudate vol. (right) (mL)	**3.72 (0.71)**	**3.59 (0.65)**	**3.61 (0.77)**	-	-	-

*MRI findings for baseline, 18 months and 36 months, both for participants included in analysis I and participants included in analysis II. Bold text in analysis I represents a thicker cortex or higher subcortical volume than in the contralateral hemisphere. eTIV, Estimated total intracranial volume; Th., Thickness; Med. Temp, Medial temporal. Lat. Temp, Lateral temporal; Vol., volume*.

Comparisons revealed no significant differences in age, sex, mRs, CCI, or frequency of diabetes in participants with strokes in left or right hemisphere (Supplementary Table 3).

### Brain Changes and Ipsilesional vs. Contralesional Stroke Hemisphere—Analysis I

Analysis I was conducted in 230 (94.3%) of the participants, with no major differences in baseline characteristics between this subgroup and the 244 of the entire study population.

As represented in bold font in Table 2, mean left hemisphere volumes and thicknesses were generally higher than the right hemisphere volumes and thicknesses at baseline, 18, and 36 months. Results across time did however show that the selected cortices and deep brain structures in the right hemisphere were generally thicker and had higher volumes, except for in the thalamus and the frontal and parietal lobes.

A thickness/volumetric decline over time was found in all ipsilesional cortices/structures, except in the left frontal lobe (Supplementary Table 4).

**Figure 2:**
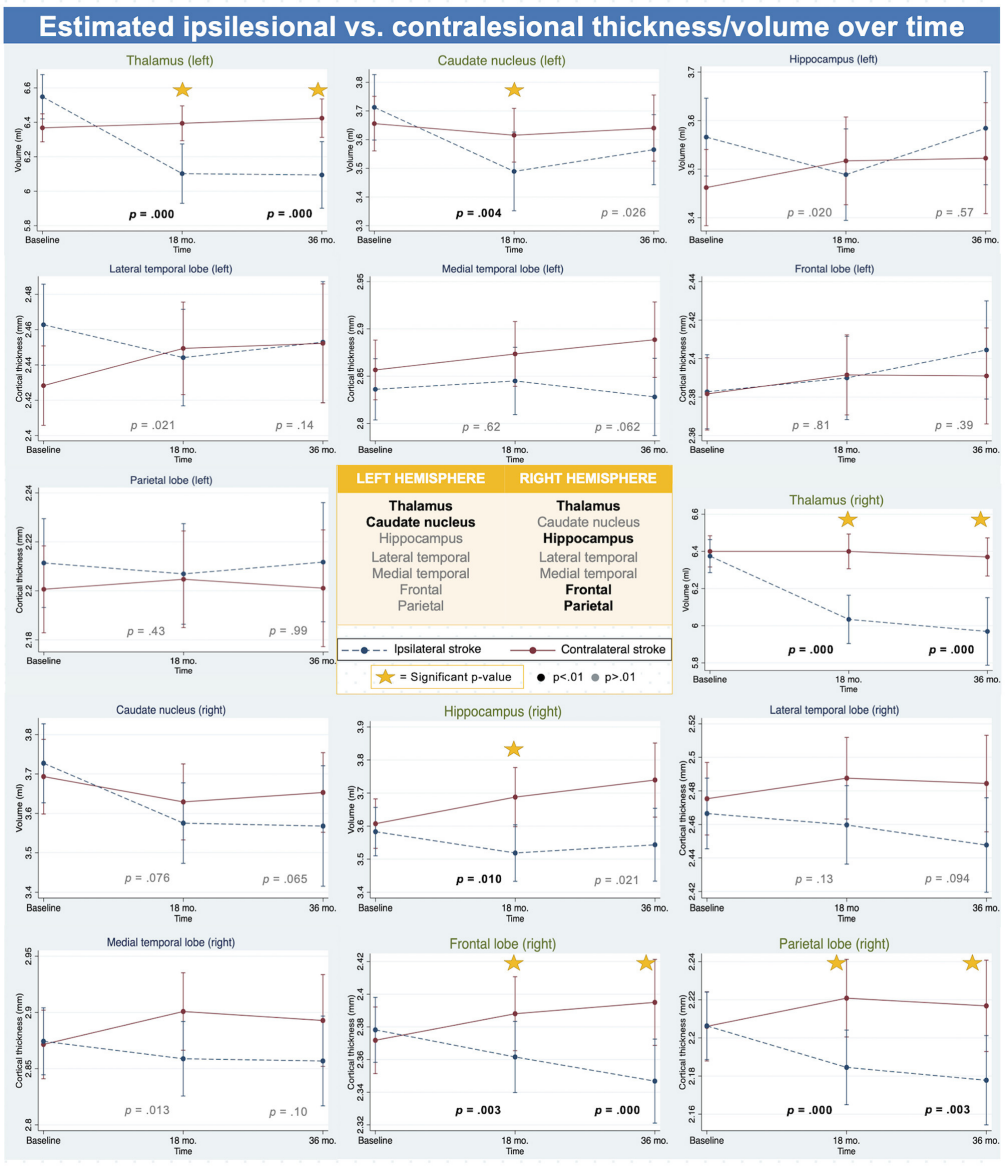
Brain atrophy by stroke hemisphere - analysis I. Figure depicting thickness and volume change over time in ipsilesional vs. contralesional hemisphere in left and right hemisphere, showing the effect was more prominent in right hemisphere areas than in the left hemisphere areas. Significant *p-*value showed in black and bold at time of result. *P-*values are for the differences in change from baseline to 18 or 36 months (interaction terms). Significant *p-*value are in black and bold. Triangle next to *p-*value indicates significant three-way interaction, indicating a difference between the hemispheres in the effect of contra vs. ipsilesional stroke at timepoint.

Estimated marginal means (Figure 2) showed that a right-sided stroke led to overall ipsilesional cortical thickness and subcortical volume loss at 18 months. On the other hand, a left-sided stroke also led to ipsilesional subcortical volume loss, but cortical loss was only found in the lateral temporal lobe. The significant findings from the linear mixed models showed a decline in thickness/volume of ipsilesional cortex/subcortical structures, often with a corresponding slight increase in the contralesional counterpart.

The linear mixed models in analysis I showed that thickness/volume decline was significantly greater in the ipsilesional hemisphere than in the contralesional hemisphere from baseline to 18 months for the right frontal- (−0.033 mm, *p* = 0.003) and parietal cortex (−0.037 mm, *p* < 0.001), the right hippocampus (−0.145 mL, *p* = 0.010), both the left and the right thalamus (−0.473 mL, *p* < 0.001; −0.340 mL, *p* < 0.001, respectively), and the left caudate (−0.183 mL, *p* = 0.004). All significant results at 18 months were also significant at 36 months, except for the right hippocampus (−0.172 mL, *p* = 0.021) and the left caudate (−0.132, *p* = 0.026), not reaching significant levels. For a complete overview of all results, see Supplementary Table 4. For all estimated marginal means, see Supplementary Table 5.

The LME models including both left and right hemispheres and its interactions with ipsilesional vs. contralesional stroke hemisphere and time, revealed significant three-way interactions for all cortices and subcortices (except the frontal lobe) at 18 months, and for the thalamus and caudate nucleus at 36 months (Supplementary Table 6). The significant three-way interactions showed that there was a significant difference in atrophy between the left and right hemisphere, dependent on whether the hemisphere was ipsilesional or contralesional to the stroke, across time.

### Cortical and Subcortical Atrophy and Cognitive Impairment—Analysis II

Analysis II was conducted in 203 (83.2%) of the entire population. This subset consisted of 55.2% women, with the age of 71.0 (11.1) years, and 12.4 (3.7) years of education (see Table 1). The only major difference in the analysis II study sample was a smaller average stroke lesion volume [8.6 (17.6) mL] than in the entire study sample group [12.2 (29.1) mL].

**Figure 3:**
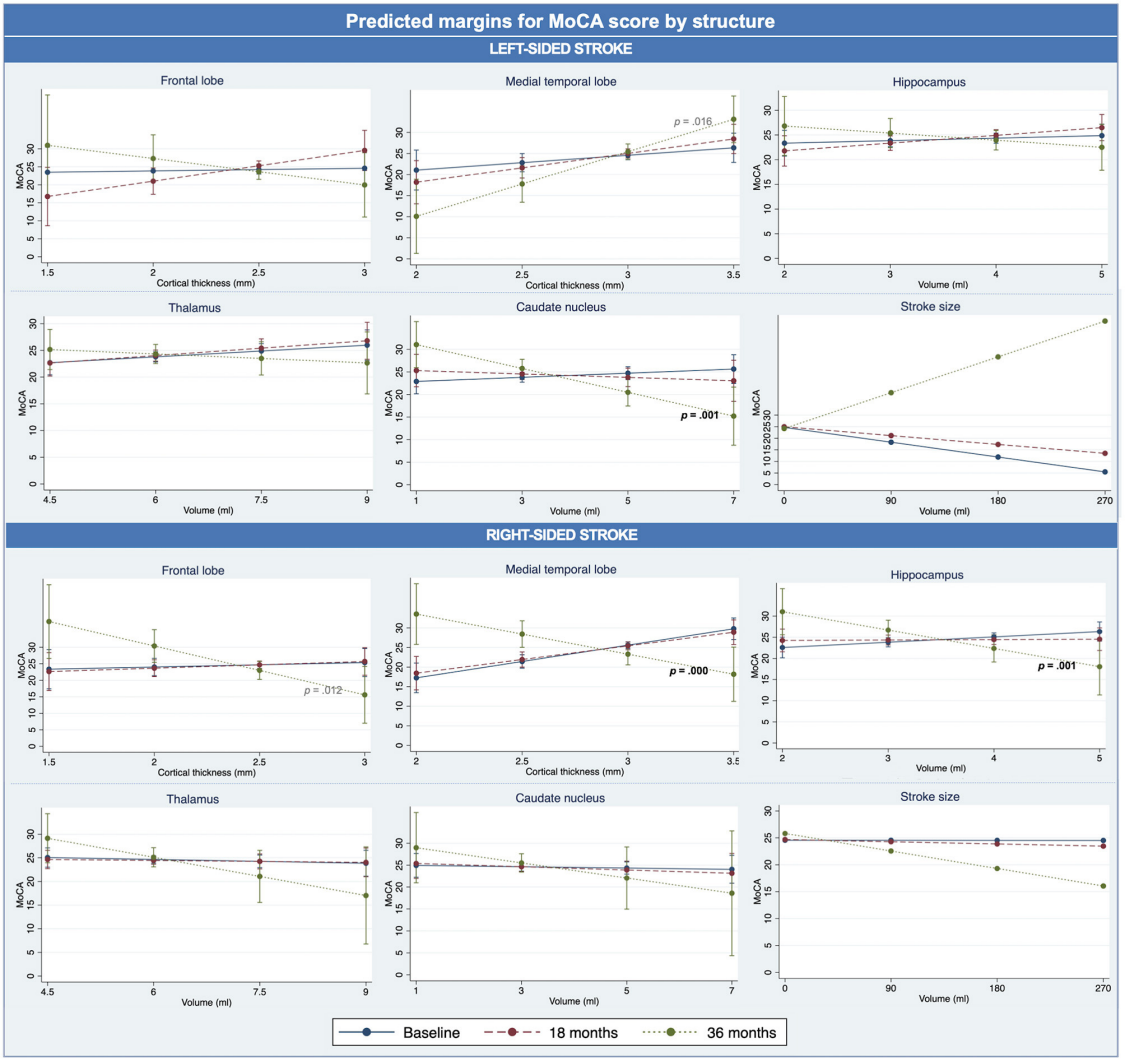
Brain changes and cognition – analysis II. Estimated marginal means for MoCA score for each structure, over time. Significant *p-*values in black bold, near significant *p-*value in gray non-bold.

Figure 3 and Supplementary Table 7 show the trends that a thicker cortex/higher subcortical volume at both baseline and 18 months was linked to higher MoCA scores. However, the only significant findings were that a thinner medial temporal lobe cortex and a lower hippocampal volume after a right-sided stroke, and lower caudate nucleus volume after a left-sided stroke, were associated with better cognition at 36 months. A smaller stroke volume was associated with better cognition over time, except at 36 months after a left-sided stroke. The associations with stroke volume were however not significant. For estimated marginal means, see Supplementary Table 8. The additional models including WMH as a covariate revealed slight changes to all models (Supplementary Table 4). The most significant changes were the medial temporal lobe after a left-sided stroke going from almost significant at the 1% level (*p* = 0.016) to becoming significant (*p* = 0.006), the medial temporal lobe after a right-sided stroke going from significant (*p* = 0.000) to not significant (*p* = 0.013), and with the hippocampus after a right-sided stroke from significant (*p* =.001) to not significant (*p* = 0.59).

## Discussion

With the use of longitudinal data from the acute stroke event up to 36 months of follow-up, we found (I) greater ipsilesional than contralesional cortical and subcortical atrophy in most cortices and deep brain structures. This difference in structure atrophy rates between the ipsilesional vs. contralesional differed between the left and right hemisphere, such that the effect was more prominent in the right hemisphere. For the right hemisphere, ipsilesional cortical thickness became increasingly lower in the frontal and parietal cortex, with similar trends observed in the medial temporal lobe and (at the trend level) the lateral temporal lobe. Overall, ipsilesional cortical atrophy was greater in all cortices in the right hemisphere except in the lateral temporal lobe, whereas for the left hemisphere a greater ipsilesional cortical atrophy was found in the lateral temporal lobe *only*. A greater ipsilesional than contralesional volume loss was found for subcortical structures also, but the differences between the hemispheres were small. Most significant results at 18 months remained significant at 36 months, although the difference in atrophy rate between the ipsilesional and contralesional hemisphere was smaller with time. Next, although generally not reaching significant levels, the overall trend showed as expected that (II) a thicker cortex or a higher subcortical structure volume, as well as a smaller stroke volume, were all associated with better cognition at baseline and 18 months. Most results at 36 months did surprisingly show the *opposite* effect, except for in the medial temporal lobe after a left-sided stroke and for stroke volume after a right-sided stroke.

Findings from analysis I revealed that the ipsilesional cortices generally atrophied more than their contralesional counterparts. Most of the findings showed a lateralization effect, such that this effect was found in almost all cortical regions of the right hemisphere after a stroke in any location of the right hemisphere, but not in all left-sided cortices after a left-sided stroke. This suggests that the right hemisphere is less resilient to a stroke in its hemisphere than the left hemisphere. Greater right-sided hemispheric atrophy has been found with healthy aging (39, 40) and a stroke in this hemisphere may exacerbate an already accelerated atrophy. Some cortical regions did however show trends toward an opposite lateralization effect, with a greater atrophy rate in the ipsilesional lateral temporal-, frontal-, and parietal lobes after a left-sided stroke. For the lateral temporal lobe, this may be due to our study cohort having lower cortical thickness in the left compared to right hemisphere in the lateral temporal cortex, perhaps leaving it less resilient to a stroke event. Lower cortical thickness in the left hemisphere was, however, not present in the frontal- and parietal lobes. Another explanation could be a dissimilarity between the hemispheres in structural plasticity after a stroke (41). Such effects are mostly studied with follow-up times only up until 1 year, and although the current findings suggest such a left-right hemispheric dissimilarity, this remains to be confirmed in other and longer lasting studies. Note that cortical thickness and subcortical volumetric asymmetry is common in healthy adults, with the left hemisphere predominantly having a thicker cortex, but a smaller surface area than the right hemisphere (42). The ENIGMA study has published results on left-right laterality in health and disease with study populations up to 17 000. Overall, these studies indicate that the left hemisphere has a thicker cortex in anterior regions (frontal lobe, primary sensory areas, superior parietal lobe, and anterior portions of the medial temporal lobe), whereas the right hemisphere has a thicker cortex in more posterior portions of the brain (lateral and medial temporal lobe, parietal lobe and occipital lobe). The same pattern was found in the current study population, except for in the parietal- and medial temporal lobes. This slight difference may be due to the ENIGMA study subdividing the lobes into more levels than the current study. The difference may however also be due to the current study population being older and burdened with both stroke lesions and pre-existing brain pathology (3), such as WMH, which are associated with lower cortical thickness (11, 43). Other studies also suggest more complex views on hemispheric asymmetry with age (44). However, being able to find almost the exact same brain structure asymmetry pattern as ENIGMA with a relatively small study sample strengthens the likelihood of this pattern to be a robust finding.

As for the subcortical structures, including the hippocampus, we found that thalamus was larger in the left hemisphere while the hippocampus and the caudate nucleus were larger in the right hemisphere. This is also in line with the findings of the ENIGMA study (42). In the current study we demonstrated that the ipsilesional subcortical structures had greater atrophy rate than their contralateral counterparts, irrespective of hemisphere, although the right caudate nucleus and the left hippocampus volume loss were only at the trend level. Greater ipsilesional atrophy has been shown in subcortical structures within the first year after a stroke (11, 14). An explanation of why all subcortical regions, in contrast to all cortical regions, showed a more pronounced ipsilesional as opposed to contralesional atrophy could simply be that many of the participants had subcortical strokes. It seems more likely though, that the subcortical structures are more vulnerable as these structures are part of many networks in the brain and thereby indirectly affected by a stroke located to any parts of this network (45). This is particularly true for thalamus, which was the structure with the most consistent effects at both 18- and 36 months. Thalamus is a relay station in the brain and is therefore connected to a high number of cortical and subcortical regions. Next, atrophy of subcortical structures, in particular hippocampal atrophy, is a hallmark manifestation of vascular risk factors and vascular dementia (46, 47). As there was a high level of vascular pathology in this patient group, a high level of atrophy of the subcortical structures was not a surprising find.

Findings from analysis II were in line with our predictions, but most were statistical trends, not reaching the predetermined significance level of p <0.01. The results implies that a thicker cortex and a larger subcortical structure volume were associated with better cognition at 18 months. The only prediction reaching a significant level showed that higher cognitive scores at 36 months were associated with a thicker medial temporal cortex after a left-sided stroke. Although not significant, another finding in line with the expected trend at 36 months was the association between a higher cognitive score and a smaller stroke lesion size after a right-sided stroke. These findings are not surprising as the left medial temporal lobe plays a fundamental role in memory and language - important aspects of cognition, but also for cognitive testing, e.g., with MoCA (48). Adding WMH to the models altered the results so that two findings were no longer significant while one became more significant, suggesting that the presence of microvascular disease in white matter affected cognitive outcome after stroke, in line with previous studies (4–6). Stroke lesion volume has been found to be an important predictor of cognitive outcome after stroke, when utilizing more comprehensive batteries of cognitive tests than in the current study (4, 5, 49). The fact that an effect of the size of the stroke was found only in the right hemisphere is in line with the results of analysis I, showing that the right hemisphere may be less resilient to an ipsilateral stroke. Taken together, stroke and WMH volumes appeared to have played a limited role for cognitive outcome across 36 months after stroke.

It is generally reported that left-sided strokes are both more frequent and associated with worse cognitive outcome (50, 51), but the current findings suggests that the cognitive consequences of a right-sided stroke merely manifest differently by maybe having a later onset than observed after a left-sided stroke. Atypical (or a reduction of) hemispheric asymmetry is reported in multiple cognitive and psychiatric disorders (42, 52). Here we showed that a right-sided stroke changed the hemispheric asymmetry more than a left-sided one. This will potentially have consequences both for degree and timing of onset of cognitive deficit. Similar to other studies, we demonstrated that the left lateral temporal lobe was more vulnerable to an ipsilateral stroke than its counterpart. This region is responsible for cognitive functions such as language comprehension, hearing and facial recognition (53). Outcome after stroke is often assessed with cognitive testing depending on left hemisphere dominant abilities, in particular language, and outcome may therefore appear disproportionally negative for a left-sided stroke (54). Our results imply that the cognitive effects of greater atrophy rates in the right hemisphere may potentially appear later than what is usually studied in stroke. Neurodegeneration is a slow process with consequences emerging later rather than sooner. Early emergence of post-stroke dementia (<3 months) after a stroke has been found to be associated with (a mix of, but) mostly vascular, rather than neurodegenerative factors (5). Most stroke studies have relatively short follow-up times relative to disease duration (between 3 months to 6 years) and MRI data is rarely acquired (55). The extended follow up in this study show that neurodegeneration after stroke may have negative cognitive consequences more long term, beyond the follow-up period in most other studies, and perhaps beyond the follow up period of the current one. Stroke hemisphere may therefore lead to a difference both in degree of and in time of onset of cognitive deficits.

Surprisingly, most results from the 36 months follow-up showed that an increase in cortical thickness or subcortical structural volume was associated with *worse* cognitive outcome. These findings point at a probable bias in the current study. These findings are likely due to the loss of participants at 36 months and therefore points at a statistical power issue rather than reflecting the reality for most stroke patients. Attrition is a typical challenge to longitudinal studies, especially when the study investigates a disease in older participants (56). The participants who remained in the study for 36 months were generally healthier and had better cognition than those lost to follow-up (see Supplementary Tables 9, 10). The association between brain structure and MoCA scores from 36 months should therefore be interpreted with caution.

Another potential limitation to this study is the fact that subcortical segmentation is especially difficult for the FreeSurfer algorithm, leading to some imprecision in the subcortical measurements. This issue is however consistent across studies using this technique and the method is found satisfactory (57). The quality control performed in the current study revealed overall acceptable subcortical segmentation. Stroke lesions also pose some challenges in cortical segmentation (58) and led to exclusion of some scans.

Next, the scarceness of significant results in Analysis II may be due to the sensitivity of the cognitive test used. The MoCA scale was developed for the detection of mild cognitive impairment in a clinical setting (35) and may not be sensitive enough for evaluating the effects of the atrophy on cognition.

Next, the study included both patients with ischemic and hemorrhagic strokes, which are generally associated with different risk factors, stroke severity and outcome (59). The total number of hemorrhagic strokes was however only 8 (3.3%) and analyses including stroke subtype was therefore not feasible. Also, the use of medication was not included in the analyses and is a limitation of this study. This was however due to lack of information on how long the participants had been on the medication.

Lastly, the current study mostly included participants with mild to moderate strokes, leaving these results less generalizable to patients suffering severe strokes. The findings from Nor-COAST data have previously been found to be comparable to the Norwegian Stroke Registry, except for those with more severe strokes (60). Future studies could prevent this selection bias through basing the study on standard clinical MRI protocols rather than introducing the need for a second (study-specific) MRI at baseline. Using automated software (FreeSurfer) for cortical segmentation in brains with larger strokes is however problematic (58) leaving this type of study for this group of patients difficult.

This study has some major strengths. First, this study utilizes a large dataset that includes thorough clinical, neuropsychological and MRI examinations at time of stroke event, 18- and 36 months after stroke. The use of longitudinal data acquired over 3 years is advantageous for detecting possible delays in the onset of cognitive decline by allowing findings of both early and late onset biomarkers. Next, this study is a multi-center study, leading to a larger number of participants and a wider range of population groups and investigator characteristics. This increases the generalizability, as well as decreases potential bias in the study, although possibly leading to lower statistical power due to increased variability.

In conclusion, atrophy in ipsilesional cortices and subcortical structures was greater than in the contralesional hemisphere, demonstrating that a stroke will affect the affected hemisphere the most. This effect was more prominent in the right hemisphere with right hemispheric stroke, pointing to the left hemisphere as potentially being more resilient to an ipsilateral stroke than what the right hemisphere is. Lastly, results imply that increased atrophy, particularly in the medial temporal lobe after a left-sided stroke, and larger stroke lesion volumes after right-sided strokes were associated with cognitive decline over time and these factors should be included in the assessment of risk of cognitive decline after stroke.

## Data Availability Statement

The raw data supporting the conclusions of this article will be made available by the authors, without undue reservation.

## Ethics Statement

The studies involving human participants were reviewed and approved by REK Nord—REK Number: 2019/397. The patients/participants provided their written informed consent to participate in this study.

## Author Contributions

EA: software, formal analysis, data curation, writing—original draft and review/editing, visualization, and project administration. SL: methodology, formal analysis consultation, and writing—review/editing. DA: software and writing—review/editing. TS: software, formal analysis, and writing—review/editing. IS: writing—review/editing and funding acquisition. MB: conceptualization, methodology, writing—review/editing, supervision, project administration, and funding acquisition. AH: conceptualization, methodology, writing—review/editing, and supervision. All authors contributed to the article and approved the submitted version.

## Funding

This study, including an extensive study visit to Indiana University School of Medicine, was generously funded by the South-Eastern Norway Regional Health Authority (Grant No. 2019061). The Nor-COAST study is funded by the Norwegian National Health Association and the Norwegian University of Science and Technology (NTNU). The funding sources had no involvement in the research process. Open access publication fees were generously funded by the University of Oslo.

## Conflict of Interest

The authors declare that the research was conducted in the absence of any commercial or financial relationships that could be construed as a potential conflict of interest.

## Publisher's Note

All claims expressed in this article are solely those of the authors and do not necessarily represent those of their affiliated organizations, or those of the publisher, the editors and the reviewers. Any product that may be evaluated in this article, or claim that may be made by its manufacturer, is not guaranteed or endorsed by the publisher.
