# Supplementary material for: Longitudinal Brain Changes After Stroke and the Association With Cognitive Decline
Source: Front Neurol. 2022 Jun 3;13:856919. doi: 10.3389/fneur.2022.856919 (PMC9204010; doi:10.3389/fneur.2022.856919)
Supplement: Supplementary file 1 [file Data_Sheet_1.docx]

**
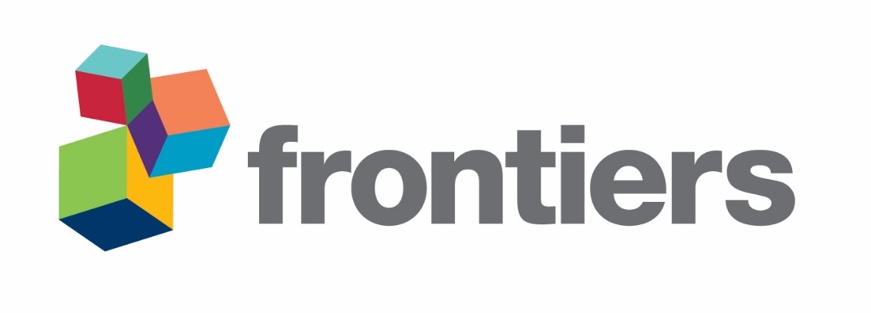
**

Supplementary Material

**Supplementary Table 1***: List of MRI sequence parameters across hospitals*

| **Hospital** | **Sequence** | **Repetition**  **Time** | **Echo**  **Time** | **Inversion**  **Time** | **Flip**  **Angle** | **Rows** | **Columns** | **FOV** | **Slice**  **Thickness** |
| --- | --- | --- | --- | --- | --- | --- | --- | --- | --- |
| Oslo | T1_BRAVO_iso | 8.16 | 3.18 | 450 | 12 | 256 | 256 | 256x256 | 1 |
|  | CUBE_FLAIR | 8000 | 125.87 | 2092 | 90 | 256 | 256 | 256x256 | 1.2 |
|  | T2-PROPELLER | 6519 | 100.9 | NA | 142 | 512 | 512 | 512x512 | 4 |
|  | SWAN_3D | 37.2 | 23.24 | NA | 10 | 512 | 512 | 512x512 | 2 |
|  | DWI_TETRA | 3500 | 61.2 | NA | 90 | 256 | 256 | 256x256 | 6 |
| St. Olav | T1_MPRAGE_iso | 2300 | 2.01 | 900 | 9 | 256 | 256 | 256x256 | 1 |
|  | 3D_FLAIR | 5000 | 388 | 1800 | 120 | 256 | 256 | 256x256 | 1 |
|  | T2_TSE | 4200 | 81 | NA | 159 | 448 | 448 | 256x256 | 3 |
|  | SWI | 29 | 30 | NA | 15 | 512 | 384 | 512x384 | 2 |
|  | DWI | 6400 | 76 | NA | 90 | 128 | 128 | 128x128 | 4 |
| Haukeland | T1_MPRAGE_iso | 2300 | 2.03 | 900 | 9 | 256 | 256 | 256x256 | 1 |
|  | 3D_FLAIR | 5000 | 386 | 1800 | 120 | 256 | 256 | 256x256 | 1 |
|  | T2 | 4370 | 73 | NA | 150 | 448 | 448 | 448x448 | 3 |
|  | SWI | 29 | 20 | NA | 15 | 512 | 384 | 512x384 | 2 |
|  | DWI | 6400 | 76 | NA | 90 | 130 | 130 | 130x130 | 4 |
| Bærum | T1-3D TFE | 7.47 | 3.44 | NA | 8 | 320 | 320 | 320x320 | 1.1 |
|  | 3D_FLAIR | 4800 | 284.1 | 1660 | 90 | 288 | 288 | 288x288 | 1.14 |
|  | T2 | 7069 | 100 | NA | 90 | 560 | 560 | 560x560 | 4 |
|  | SWI | 51 | 0 | NA | 20 | 672 | 672 | 672x672 | 2 |
|  | DWI | 4773 | 101 | NA | 90 | 224 | 224 | 224x224 | 4 |
| Ålesund | T1_3D MPRAGE (ADNI) | 25 | 4.602 | NA | 30 | 320 | 320 | 320x320 | 1 |
|  | 3D_FLAIR | 4800 | 250.5 | 1660 | 90 | 432 | 432 | 432x432 | 1.32 |
|  | T2 | 5463 | 100 | NA | 90 | 560 | 560 | 560x560 | 5 |
|  | VEN_BOLD | 24.56 | 34.7 | NA | 10 | 512 | 512 | 512x512 | 1 |
|  | DWI | 4623.9 | 108.3 | NA | 90 | 176 | 176 | 176x176 | 4 |
|  |  |  |  |  |  |  |  |  |  |

Supp. table 1: MRI sequences across the five different hospitals. All scanners used T1 3D
 gradient echo pulse sequences in order to ensure as similar image-quality as possible across hospitals. FOV = Field of view.

**Supplementary table 2***: Stroke characteristics per analysis and timepoint*

|  | **Analysis I** | | | **Analysis II** | | | |
| --- | --- | --- | --- | --- | --- | --- | --- |
|  | **PARTICIPANTS WITH UNILATERAL STROKE** | | | **PARTICIPANTS WITH COGNITIVE SCORES** | | | |
| **Variable** | **Baseline** | **18 months** | **36 months** | **Baseline** | **18 months** | | **36 months** |
| *N* | *230* | *124* | *63* | *208* | *123* | | *23* |
| ***Stroke characteristics*** | | | | | | | |
| Stroke hemisphere - right | 118 (51.3%) | 63 (50.8%) | 32 (50.8%) | 108 (51.7%) | 70 (51.9%) | 11 (42.3%) | |
| Stroke hemisphere – left | 112 (48.7%) | 61 (49.2%) | 31 (49.2%) | 95 (45.4%) | 60 (44.4%) | 14 (53.9%) | |
| Stroke hemisphere - both | - | - | - | 6 (2.9%) | 5 (3.7%) | 1 (3.8%) | |
| Stroke lobe - frontal | 63 (27.8%) | 37 (30.3%) | 21 (33.3%) | 61 (29.5%) | 39 (32%) | 7 (29.2%) | |
| Stroke lobe - subcortical | 48 (21.1%) | 25 (20.5%) | 15 (23.8%) | 44 (21.3%) | 24 (19.7%) | 5 (20.8%) | |
| Stroke lobe - parietal | 29 (12.8%) | 16 (13.1%) | 8 (12.7%) | 25 (12.1%) | 17 (13.9%) | 4 (16.7%) | |
| Stroke lobe - cerebellum | 23 (10.1%) | 11 (9.0%) | 8 (12.7%) | 22 (10.6%) | 11 (9.0%) | 5 (20.8%) | |
| Stroke lobe - occipital | 23 (10.1%) | 11 (9.0%) | 2 (3.2%) | 20 (9.7%) | 9 (7.4%) | 0 (0.0%) | |
| Stroke lobe - brainstem | 20 (8.8%) | 12 (9.8%) | 7 (11.1%) | 18 (8.7%) | 12 (9.8%) | 2 (8.3%) | |
| Stroke lobe - temporal | 13 (5.7%) | 6 (4.9%) | 1 (1.6%) | 10 (4.8%) | 6 (4.9%) | 1 (4.2%) | |
| Stroke lobe - limbic | 8 (3.5%) | 4 (3.3%) | 1 (1.6%) | 7 (3.4%) | 4 (3.3%) | 0 (0.0%) | |

*Supp. table 2: Stroke hemisphere visually detected using DWI. Stroke lobe of principal stroke, using the Talairach structural atlas (or using Harvard-Oxford atlas in 17 subjects)*

**Supplementary table 3** *– Stroke hemisphere comparisons*

| *Left vs right stroke hemisphere comparisons* | | | | | |
| --- | --- | --- | --- | --- | --- |
|  | **Left hemisphere stroke** | | **Right hemisphere stroke** | |  |
|  | *M* | *SD* | *M* | *SD* | *p* |
| Stroke volume | 12.59 | 35.42 | 11.75 | 22.44 | .83 |
| Age | 72.48 | 10.68 | 72.20 | 11.99 | .85 |
| Disability (mRs) | 2.24 | 1.25 | 2.24 | 1.32 | .99 |
| Charlson comorbidity (CCI) | 3.94 | 2.05 | 4.01 | 1.98 | .61 |
|  | *n* | *%* | *n* | *%* | *p* |
| Sex |  |  |  |  | .98 |
| Women | 65 | 56.03% | 67 | 55.83% |  |
| Men | 51 | 43.97% | 53 | 44.17% |  |
| Diabetes mellitus | 19 | 44.22% | 26 | 57.78% | .30 |
| Hypertension | 52 | 45.61% | 62 | 54.39% | .29 |
| Smoking |  |  |  |  | .65 |
| Never | 46 | 49.46% | 47 | 50.54% |  |
| Smoker | 25 | 53.19% | 22 | 46.81% |  |
| Ex-smoker | 44 | 46.32% | 51 | 53.68% |  |

*Comparisons of potential confounding variables between individuals with left vs. right hemispheric stroke. Means, SD, n or %, and p-values from t-tests or chi-square tests. mRs = modified rankin scale. CCI = Charlson comorbidity*

**Supplementary table 4:** *Analysis I. Volume/thickness by stroke hemisphere*

Supp. table 4: Adjusted vs. unadjusted linear mixed model, with the dependent variable of cortical thickness or volume per hemisphere and the covariates of ipsilesional vs. contralesional hemisphere, time-point and their interactions. Adjusted for age, sex and scanner (and etiv if dependent variable was volumetric). * = Bootstrapped #1000 in adjusted models, not in unadjusted (no important change in significance levels due to bootstrapping).

| **Ipsilesional**  **thickness/volume**  **(mm/mL)** | **ADJUSTED** | | | | | | **NON-ADJUSTED** | | | | | | |
| --- | --- | --- | --- | --- | --- | --- | --- | --- | --- | --- | --- | --- | --- |
|  | **18 months** | | | **36 months** | | | **18 months** | | | **36 months** | | | |
|  | Coef. | CI | *p* | Coef. | CI | *p* | Coef. | CI | *p* | Coef. | CI | *p* |  |
| Frontal (left) | -.003 | -.024 to .019 | .81 | .012 | -.016 to .041 | .39 | -.001 | -.023 to .021 | .92 | .013 | -.016 to .042 | .37 |  |
| Frontal (right) | **-.033** | **-.054 to -.011** | **.003** | **-.055** | **-.083 to -.026** | **.000** | **-.034** | **-.055 to -.012** | **.002** | **-.055** | **-.084 to -.027** | **.000** |  |
| Medial temporal (left) | -.008 | -.040 to .024 | .62 | -.040 | -.082 to .002 | .062 | -.006 | -.038 to .026 | .72 | -.041 | -.083 to .001 | .058 |  |
| Medial temporal (right) | -.045 | -.080 to -.010 | .013 | -.039 | -.085 to .008 | .10 | -.046 | -.081 to -.010 | .012 | -.036 | -.083 to .010 | .13 |  |
| Lateral temporal (left) | -.040 | -.073 to -.006 | .021 | -.034 | -.078 to .011 | .14 | -.036 | -.070 to -.002 | .037 | -.031 | -.075 to .014 | .18 |  |
| Lateral temporal (right) | -.019 | -.044 to .006 | .13 | -.028 | -.060 to .005 | .094 | -.020 | -.045 to .005 | .11 | -.027 | -.059 to .006 | .11 |  |
| Parietal (left) | -.009 | -.030 to .013 | .43 | -.001 | -.028 to .028 | .99 | -.007 | -.028 to .014 | .54 | .002 | -.026 to .029 | .91 |  |
| Parietal (right) | **-.037** | **-.057 to -.016** | **.000** | **-.039** | **-.066 to -.013** | **.003** | **-.038** | **-.059 to -.018** | **.000** | **-.040** | **-.067 to -.014** | **.003** |  |
| Hippocampus (left) | -.132 | -.244 to -.021 | .020 | -.042 | -.189 to .104 | .57 | -.116 | -.230 to -.001 | .047 | -.056 | -.206 to .095 | .47 |  |
| Hippocampus (right) | **-.145** | **-.256 to -.035** | **.010** | -.172 | -.317 to -.026 | .021 | **-.155** | **-.269 to -.041** | **.008** | -.157 | -.307 to -.007 | .040 |  |
| Thalamus (left)* | **-.473** | **-.668 to -.277** | **.000** | **-.510** | **-.754 to -.266** | **.000** | **-.454** | **-.637 to -.272** | **.000** | **-.547** | **-.786 to -.307** | **.000** |  |
| Thalamus (right)* | **-.340** | **-.464 to -.217** | **.000** | **-.375** | **-.566 to -.185** | **.000** | **-.346** | **-.475 to -.217** | **.000** | **-.353** | **-.523 to -.184** | **.000** |  |
| Caudate nucleus (left)* | **-.183** | **-.308 to -.058** | **.004** | -.132 | -.248 to -.016 | .026 | **-.182** | **-.289 to -.076** | **.001** | -.136 | -.276 to .004 | .057 |  |
| Caudate nucleus (right)* | -.088 | -.184 to .009 | .076 | -.119 | -.245 to .007 | .065 | -.090 | -.184 to .004 | .061 | -.115 | -.238 to .008 | .067 |  |


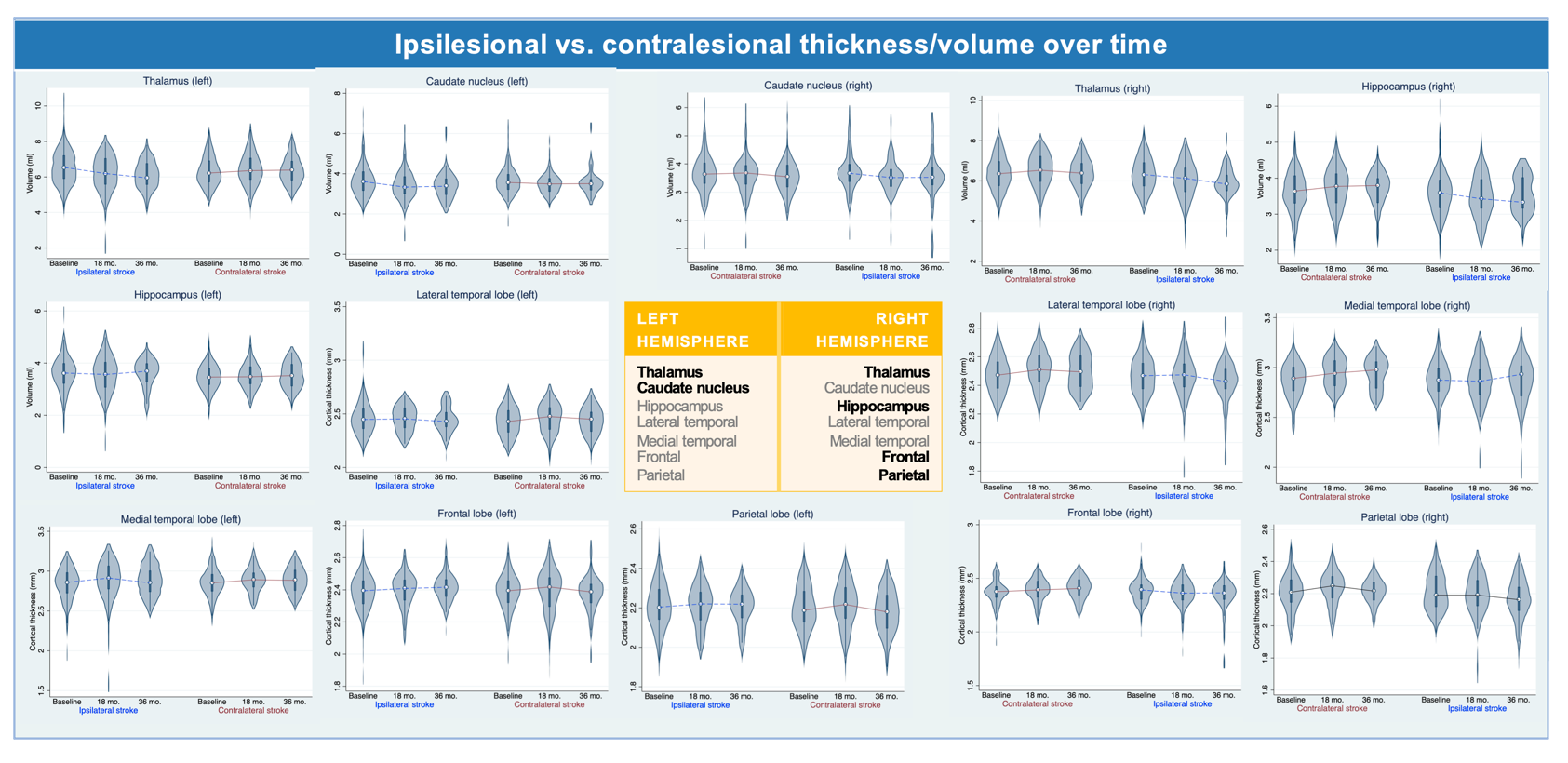


**Supplementary Figure 1:** Brain changes by stroke hemisphere (observed measurements) - analysis I

*Supp figure 1: Violin plot showing the density distributions of thickness and volume over time in ipsilesional and contralesional hemisphere in left and right hemisphere. The bold vertical bars represent the interquartile range, the non-bold vertical bars represent the 1.5x interquartile range, and the circles with connecting lines represent the median.*

|  |  | | | | | |
| --- | --- | --- | --- | --- | --- | --- |
| **Supplementary table 5:** *Analysis I. Estimated marginal means of ipsi- vs. contralesional hemisphere* | | | | | | |
| **ESTIMATED MARGINAL MEANS** | **ADJUSTED** | | | | | |
| **Volume/thickness (mm/mL)** | **Baseline** | | **18 months** | | **36 months** | |
|  | Estimate | CI | Estimate | CI | Estimate | CI |
| **Frontal (left)** |  |  |  |  |  |  |
| – Left stroke | 2.383 | 2.364 to 2.402 | 2.390 | 2.368 to 2.412 | 2.404 | 2.379 to 2.430 |
| – Right stroke | 2.382 | 2.363 to 2.400 | 2.392 | 2.371 to 2.412 | 2.391 | 2.366 to 2.416 |
| **Frontal (right)** |  |  |  |  |  |  |
| – Left stroke | **2.372** | **2.351 to 2.392** | **2.388** | **2.365 to 2.411** | **2.395** | **2.369 to 2.421** |
| – Right stroke | **2.378** | **2.358 to 2.398** | **2.363** | **2.340 to 2.383** | **2.347** | **2.321 to 2.373** |
| **Medial temporal (left)** |  |  |  |  |  |  |
| – Left stroke | 2.836 | 2.804 to 2.868 | 2.845 | 2.809 to 2.881 | 2.828 | 2.787 to 2.869 |
| – Right stroke | 2.857 | 2.825 to 2.888 | 2.874 | 2.839 to 2.908 | 2.889 | 2.849 to 2.928 |
| **Medial temporal (right)** |  |  |  |  |  |  |
| – Left stroke | 2.872 | 2.841 to 2.902 | 2.901 | 2.866 to 2.935 | 2.893 | 2.852 to 2.934 |
| – Right stroke | 2.874 | 2.845 to 2.904 | 2.859 | 2.826 to 2.892 | 2.857 | 2.817 to 2.897 |
| **Lateral temporal (left)** |  |  |  |  |  |  |
| – Left stroke | 2.463 | 2.440 to 2.486 | 2.444 | 2.417 to 2.472 | 2.453 | 2.419 to 2.487 |
| – Right stroke | 2.428 | 2.406 to 2.451 | 2.449 | 2.423 to 2.476 | 2.452 | 2.419 to 2.486 |
| **Lateral temporal (right)** |  |  |  |  |  |  |
| – Left stroke | 2.475 | 2.454 to 2.497 | 2.488 | 2.463 to 2.512 | 2.484 | 2.456 to 2.513 |
| – Right stroke | 2.467 | 2.445 to 2.488 | 2.460 | 2.436 to 2.483 | 2.448 | 2.420 to 2.476 |
| **Parietal (left)** |  |  |  |  |  |  |
| – Left stroke | 2.211 | 2.193 to 2.230 | 2.207 | 2.186 to 2.227 | 2.212 | 2.187 to 2.236 |
| – Right stroke | 2.201 | 2.183 to 2.218 | 2.205 | 2.185 to 2.224 | 2.201 | 2.177 to 2.225 |
| **Parietal (right)** |  |  |  |  |  |  |
| – Left stroke | **2.206** | **2.188 to 2.224** | **2.221** | **2.201 to 2.241** | **2.217** | **2.193 to 2.241** |
| – Right stroke | **2.206** | **2.189 to 2.224** | **2.185** | **2.165 to 2.204** | **2.178** | **2.154 to 2.201** |
| **Hippocampus (left)** |  |  |  |  |  |  |
| – Left stroke | 3.566 | 3.486 to 3.646 | 3.489 | 3.395 to 3.583 | 3.584 | 3.468 to 3.701 |
| – Right stroke | 3.462 | 3.384 to 3.541 | 3.517 | 3.427 to 3.607 | 3.523 | 3.408 to 3.637 |
| **Hippocampus (right)** |  |  |  |  |  |  |
| – Left stroke | **3.607** | **3.533 to 3.682** | **3.688** | **3.599 to 3.777** | **3.739** | **3.627 to 3.852** |
| – Right stroke | **3.583** | **3.510 to 3.657** | **3.519** | **3.433 to 3.604** | **3.544** | **3.434 to 3.654** |
| **Thalamus (left)*** |  |  |  |  |  |  |
| – Left stroke | **6.549** | **6.420 to 6.677** | **6.102** | **5.930 to 6.274** | **6.094** | **5.901 to 6.288** |
| – Right stroke | **6.368** | **6.287 to 6.449** | **6.394** | **6.293 to 6.495** | **6.424** | **6.313 to 6.536** |
| **Thalamus (right)*** |  |  |  |  |  |  |
| – Left stroke | **6.400** | **6.316 to 6.484** | **6.400** | **6.307 to 6.493** | **6.370** | **6.267 to 6.473** |
| – Right stroke | **6.375** | **6.286 to 6.464** | **6.034** | **5.904 to 6.164** | **5.969** | **5.788 to 6.151** |
| **Caudate nucleus (left)*** |  |  |  |  |  |  |
| – Left stroke | **3.713** | **3.598 to 3.827** | **3.489** | **3.352 to 3.626** | **3.565** | **3.443 to 3.687** |
| – Right stroke | **3.656** | **3.561 to 3.751** | **3.615** | **3.522 to 3.709** | **3.640** | **3.525 to 3.756** |
| **Caudate nucleus (right)*** |  |  |  |  |  |  |
| – Left stroke | 3.693 | 3.599 to 3.788 | 3.629 | 3.533 to 3.725 | 3.653 | 3.552 to 3.754 |
| – Right stroke | 3.727 | 3.627 to 3.827 | 3.575 | 3.473 to 3.678 | 3.568 | 3.415 to 3.721 |

*Supp. table 5: Estimated marginal means from the linear mixed model, with the dependent variable of cortical thickness or volume per hemisphere and the covariates of ipsilesional vs. contralesional hemisphere, time-point and their interactions. Adjusted for age, sex and scanner (and etiv if dependent variable was volumetric). Numbers in bold correspond to significant results in the linear regression model.*

**Supplementary table 6 *–*** *Linear mixed model analyses with three-way interaction as described under analysis I*

| **Three-way interactions** | | |
| --- | --- | --- |
|  | 18 months | 36 months |
| Thalamus | ***p* <.001** | ***p* <.001** |
| Caudate nucleus | ***p =* .007** | ***p =* .007** |
| Hippocampus | ***p* <.001** | *p* ***=*** .078 |
| Lateral temporal | ***p =* .001** | *p* ***=*** .16 |
| Medial temporal | ***p =* .010** | *p* ***=*** .12 |
| Frontal | *p* ***=*** .014 | *p* ***=*** .079 |
| Parietal | ***p =* .001** | *p* ***=*** .025 |

*p-values for three-way interactions between contra vs. Ipsilesional cortical thickness or subcortical volume, time, and stroke hemisphere, per thickness/volume.*

**Supplementary table 7:** Results of analysis II: Cortical thicknesses/subcortical volumes and cognition

| ***Volume/thickness*** | **PRIMARY ANALYSES (without WMH)** | | | | | | **ADDITIONAL ANALYSES (with WMH)** | | | | | |
| --- | --- | --- | --- | --- | --- | --- | --- | --- | --- | --- | --- | --- |
|  | **18 months** | | | **36 months** | | | **18 months** | | | **36 months** | | |
| *(mm/mL)* | Coef. | CI | *p* | Coef. | CI | *p* | Coef. | CI | *p* | Coef. | CI | *p* |
| **Frontal** |  |  |  |  |  |  |  |  |  |  |  |  |
| – left stroke | 7.80 | -1.25 to 16.85 | .091 | 19.65 | -17.71 to 57.00 | .30 | 7.54 | -2.27 to 17.36 | .13 | -12.87 | -29.79 to 4.05 | .13 |
| – right stroke | .70 | -5.81 to 7.21 | .83 | -16.1 | -29.37 to -2.91 | .012 | 2.51 | -4.53 to 9.56 | .48 | -31.09 | -89.56 to 27.39 | .30 * |
| **Medial temporal** |  |  |  |  |  |  |  |  |  |  |  |  |
| – left stroke | 3.32 | -24.80 to 5.74 | .22 | 11.83 | 2.25 to 21.41 | .016 | 2.42 | -3.06 to 7.89 | .39 | **-41.50** | **-71.25 to -11.75** | **.006** |
| – right stroke | -1.38 | -6.32 to 3.56 | .58 | **-18.61** | **-28.29 to -8.93** | **.000** | -.65 | -6.27 to 4.97 | .82 | -15.38 | -27.46 to -3.29 | .013 |
| **Hippocampus** |  |  |  |  |  |  |  |  |  |  |  |  |
| – left stroke | 1.06 | -.49 to 2.62 | .21 | -1.95 | -5.12 to 1.22 | .23 | .81 | -.92 to 2.43 | .33 | -2.09 | -5.34 to 1.16 | .21 |
| – right stroke | -1.16 | -2.73 to .41 | .14 | **-5.59** | **-9.39 to -1.80** | **.001** | -1.53 | -3.20 to .13 | .071 | -3.89 | -7.93 to .15 | .059* |
| **Thalamus** |  |  |  |  |  |  |  |  |  |  |  |  |
| – left stroke | .19 | -.90 to 1.29 | .73 | -1.28 | -3.16 to .59 | .18 | .18 | -.96 to 1.31 | .76 | -1.53 | -3.53 to .48 | .14 |
| – right stroke | .13 | -.87 to 1.13 | .80 | -2.42 | -5.67 to .82 | .14 | -.03 | -1.10 to 1.03 | .95 | -1.68 | -4.91 to 1.56 | .31* |
| **Caudate nucleus** |  |  |  |  |  |  |  |  |  |  |  |  |
| – left stroke | -.83 | -1.95 to -.28 | .12 | **-3.10** | **-4.83 to -1.37** | **.001** | -.46 | -1.65 to .73 | .45 | **-3.00** | **-4.77 to -1.23** | **.001** |
| – right stroke | -.22 | -1.51 to 1.06 | .74 | -1.58 | -5.27 to 2.11 | .24 | -.63 | -1.96 to .71 | .36 | .62 | -5.59 to 6.93 | .85* |
| **Stroke size** |  |  |  |  |  |  |  |  |  |  |  |  |
| – left stroke | .03 | -.06 to .11 | .51 | .24 | -.19 to .68 | .27 | .03 | -.06 to .11 | .55 | .29 | -.16 to .74 | .21 |
| – right stroke | -.01 | -.05 to .04 | .84 | -.04 | -.14 to .07 | .49 | .01 | -.04 to .04 | 1.00 | .01 | -.09 to .12 | .80* |

Supp. table 7: Left side shows results from the adjusted linear mixed models, with dependent variable of cognition and the covariates of cortical thickness or subcortical volume, stroke hemisphere, time-point and their interactions. Adjusted for age, sex, scanner and education. Right side shows same models with added WMH as covariate.

**Supplementary table 8:** Analysis II. Estimated marginal means of cognition (MoCA-scores)

| **ESTIMATED MARGINAL MEANS** |  |  |  |  |  |  |
| --- | --- | --- | --- | --- | --- | --- |
|  | **ADJUSTED** | | | | | |
| **Volume/thickness** | **Baseline** | | **18 months** | | **36 months** | |
|  | Estimate | CI | Estimate | CI | Estimate | CI |
| **Frontal lobe (left stroke)** |  |  |  |  |  |  |
| - 1.5 mm | 23.5 | 16.5 to 30.5 | 16.7 | 8.6 to 24.9 | 31.0 | 17.0 to 45.0 |
| - 2 mm | 23.9 | 20.8 to 27.0 | 21.0 | 17.4 to 24.7 | 27.3 | 20.7 to 33.9 |
| - 2.5 mm | 24.2 | 23.0 to 25.5 | 25.3 | 23.9 to 26.6 | 23.6 | 21.5 to 25.8 |
| - 3 mm | 24.6 | 19.6 to 29.6 | 29.6 | 24.0 to 35.1 | 20.0 | 11.0 to 28.9 |
| **Frontal lobe (right stroke)** |  |  |  |  |  |  |
| - 1.5 mm | 23.4 | 17.4 to 29.3 | 22.6 | 16.9 to 28.4 | 37.8 | 26.6 to 48.9 |
| - 2 mm | 24.0 | 21.4 to 26.7 | 23.7 | 21.1 to 26.2 | 30.4 | 25.5 to 35.3 |
| - 2.5 mm | 24.7 | 23.6 to 25.8 | 24.7 | 23.5 to 25.8 | 23.0 | 20.3 to 25.7 |
| - 3 mm | 25.4 | 21.1 to 29.6 | 25.7 | 21.5 to 29.9 | 15.6 | 7.0 to 24.2 |
| **Medial temporal (left stroke)** |  |  |  |  |  |  |
| - 2 mm | 21.1 | 16.3 to 25.8 | 18.2 | 13.0 to 23.3 | 10.1 | 1.3 to 18.9 |
| - 2.5 mm | 22.8 | 20.7 to 25.0 | 21.6 | 19.2 to 24.0 | 17.8 | 13.4 to 22.1 |
| - 3 mm | 24.6 | 23.5 to 25.6 | 25.0 | 24.0 to 26.1 | 25.4 | 23.6 to 27.3 |
| - 3.5 mm | 26.4 | 22.9 to 29.8 | 28.5 | 25.0 to 31.9 | 33.1 | 27.6 to 38.7 |
| **Medial temporal (right stroke)** |  |  |  |  |  |  |
| - 2 mm | **17.2** | **13.5 to 21.0** | **18.4** | **14.2 to 22.7** | **33.6** | **25.8 to 41.3** |
| - 2.5 mm | **21.4** | **19.7 to 23.1** | **21.9** | **20.0 to 23.9** | **28.4** | **25.0 to 31.8** |
| - 3 mm | **25.6** | **24.8 to 26.5** | **25.4** | **24.4 to 26.4** | **23.3** | **20.6 to 26.0** |
| - 3.5 mm | **29.8** | **27.0 to 32.5** | **28.9** | **25.8 to 32.1** | **18.2** | **11.2 to 25.1** |
| **Hippocampus (left stroke)** |  |  |  |  |  |  |
| - 2 mL | 23.3 | 20.7 to 26.0 | 21.8 | 18.7 to 24.8 | 26.8 | 20.8 to 32.8 |
| - 3 mL | 23.9 | 22.6 to 25.1 | 23.3 | 21.9 to 24.8 | 25.4 | 22.4 to 28.3 |
| - 4 mL | 24.4 | 23.3 to 25.4 | 24.9 | 23.8 to 26.1 | 23.9 | 22.0 to 25.9 |
| - 5 mL | 24.9 | 22.5 to 27.2 | 26.5 | 23.8 to 29.1 | 22.5 | 17.9 to 27.1 |
| **Hippocampus (right stroke)** |  |  |  |  |  |  |
| - 2 mL | **22.6** | **20.2 to 25.0** | **24.3** | **21.6 to 26.9** | **31.0** | **25.6 to 36.5** |
| - 3 mL | **23.8** | **22.8 to 24.9** | **24.4** | **23.2 to 25.5** | **26.7** | **24.4 to 29.0** |
| - 4 mL | **25.1** | **24.2 to 26.0** | **24.5** | **23.3 to 25.6** | **22.4** | **19.1 to 25.6** |
| - 5 mL | **26.4** | **24.1 to 28.6** | **24.6** | **21.9 to 27.2** | **18.0** | **11.4 to 24.7** |
| **Thalamus (left stroke)** |  |  |  |  |  |  |
| - 4.5 mL | 22.7 | 20.4 to 25.0 | 22.7 | 20.2 to 25.1 | 25.2 | 21.4 to 28.9 |
| - 6 mL | 23.8 | 22.8 to 24.8 | 24.0 | 23.0 to 25.1 | 24.3 | 22.6 to 26.1 |
| - 7.5 mL | 24.9 | 23.5 to 26.3 | 25.4 | 23.7 to 27.2 | 23.5 | 20.4 to 26.6 |
| - 9 mL | 26.0 | 23.1 to 28.8 | 26.8 | 23.3 to 30.3 | 22.7 | 16.9 to 28.5 |
| **Thalamus (right stroke)** |  |  |  |  |  |  |
| - 4.5 mL | 25.0 | 23.0 to 27.1 | 24.6 | 22.7 to 26.6 | 29.1 | 24.0 to 34.3 |
| - 6 mL | 24.7 | 23.9 to 25.4 | 24.4 | 23.6 to 25.3 | 25.1 | 23.1 to 27.1 |
| - 7.5 mL | 24.3 | 22.9 to 25.6 | 24.3 | 22.7 to 25.8 | 21.1 | 15.6 to 26.6 |
| - 9 mL | 23.9 | 21.1 to 26.6 | 24.0 | 21.0 to 27.1 | 17.0 | 6.8 to 27.3 |
| **Caudate nucleus (left stroke)** |  |  |  |  |  |  |
| - 1 mL | **22.9** | **20.2 to 25.6** | **25.3** | **21.7 to 28.9** | **31.0** | **25.9 to 36.2** |
| - 3 mL | **23.8** | **22.7 to 24.9** | **24.5** | **23.3 to 25.8** | **25.8** | **23.7 to 27.8** |
| - 5 mL | **24.7** | **23.3 to 26.1** | **23.8** | **21.7 to 25.8** | **20.5** | **17.4 to 23.5** |
| - 7 mL | **25.6** | **22.4 to 28.8** | **23.0** | **18.5 to 27.6** | **15.2** | **8.8 to 21.6** |
| **Caudate nucleus (right stroke)** |  |  |  |  |  |  |
| - 1 mL | 24.9 | 22.2 to 27.7 | 25.4 | 22.0 to 28.8 | 29.0 | 21.0 to 37.0 |
| - 3 mL | 24.6 | 23.7 to 25.6 | 24.7 | 23.6 to 25.7 | 25.5 | 23.4 to 27.6 |
| - 5 mL | 24.3 | 23.0 to 25.7 | 23.9 | 21.9 to 25.9 | 22.1 | 15.0 to 29.1 |
| - 7 mL | 24.0 | 20.9 to 27.2 | 23.2 | 18.6 to 27.7 | 18.6 | 4.4 to 32.8 |
| **Stroke size (left stroke)** |  |  |  |  |  |  |
| - 0.01 mL | 24.7 | 23.8 to 25.6 | 25.0 | 23.9 to 26.1 | 24.2 | 22.1 to 26.3 |
| - 90 mL | 18.3 | 11.8 to 24.8 | 21.2 | 14.2 to 28.1 | 39.7 | 1.9 to 77.5 |
| - 180 mL | 11.9 | -1.4 to 25.2 | 17.3 | 3.1 to 31.6 | 55.2 | -21.6 to 132.0 |
| - 270 mL | 5.5 | -14.6 to 25.6 | 13.5 | -8.1 to 35.1 | 70.7 | -45.1 to 186.5 |
| **Stroke size (right stroke)** |  |  |  |  |  |  |
| - 1 mL | 24.6 | 23.8 to 25.3 | 24.7 | 23.7 to 25.6 | 25.8 | 23.0 to 28.6 |
| - 3 mL | 24.5 | 21.9 to 27.1 | 24.3 | 20.8 to 27.7 | 22.6 | 14.8 to 30.3 |
| - 5 mL | 24.5 | 19.1 to 30.0 | 23.9 | 16.7 to 31.1 | 19.3 | 2.4 to 36.2 |
| - 7 mL | 24.5 | 16.2 to 32.8 | 23.5 | 12.5 to 34.4 | 16.0 | -10.2 to 42.3 |

*Supp. table 8: Estimated marginal means from the linear mixed model, with dependent variable of cognition and covariates of cortical thickness or volume, stroke hemisphere, time-point and their interactions. Adjusted for age, sex, scanner and education. Numbers in bold correspond to significant results in the linear regression model.*

**Supplementary Table 9***: Characteristics of participants with MRI data at 1, 2, or 3 timepoints*

|  | **ONLY 1 TIMEPOINT** | **2 TIMEPOINTS** | **3 TIMEPOINTS** |  |
| --- | --- | --- | --- | --- |
| *N* | *161* | *203* | *72* |  |
| Women | 50.3 % | 57.6% | 58.3% | .313 |
| **Age** | **73.0 (12.6)** | **70.4 (10.7)** | **71.1 (8.5)** | **.001** |
| Education (years) | 12.3 (3.9) | 12.6 (3.7) | 12.5 (3.9) | .798 |
| **GDS (pre-stroke)** | **1.59 (1.01)** | **1.27 (.59)** | **1.18 (.39)** | **.000** |
| **mRS** | **2.3 (1.4)** | **1.9 (1.2)** | **1.8 (1.0)** | **.002** |
| **Comorbidity (CCI)** | **4.1 (2.0)** | **3.7 (1.9)** | **3.5 (1.5)** | **.021** |
| NIHSS | 4.3 (4.9) | 3.7 (4.9) | 4.1 (5.9) | .100 |
| **MoCA** | **23.3 (5.6)** | **25.1 (4.2)** | **26.0 (4.2)** | **.000** |
| **NCD normal** | **39.1%** | **53.6%** | **59.1%** | **.000** |
| **NCD mild** | **24.2%** | **31.1%** | **26.8%** |  |
| **NCD major** | **36.7%** | **15.3%** | **14.1%** |  |
| **Stroke size (mL)** | **15.53 (31.86)** | **9.81 (26.58)** | **8.10 (18.58)** | **.000** |
| Med temp thick (mm) | 2.82 (.17) | 2.91 (.17) | 2.94 (.13) | .188 |

*Supp. Table 6: p-values from one way ANOVA or Pearson’s chi squared test presented in order to show substantial difference between groups. NCD and mRS from baseline. (N) = Data missing. Significant differences in bold, with healthier with 3 timepoints in yellow frame. GDS = Global deterioration scale. mRS = modified Rankin Scale. NIHSS = National Institutes of Health Stroke Scale. MoCA = Montreal Cognitive Assessment. NCD = Neurocognitive disorder.*

*Frailty:* ***0-4.*** *Fatigue + stairs + walk + illnesses + weight loss.*

*GDS:* ***1-7****. 2-3 = MCI. 4-7 = Dementia.*

*mRS:* ***0-6.***

*CCI:* ***0-29****.*

*NIHSS:* ***0-42****.*

*MoCA:* ***0-30.***

|  | **18 🡪 36 months**  **Supplementary Table 10:** *Characteristics of 18-36 months non drop-outs vs. drop-outs* | **18 🡪 36 months**  **drop-outs** |
| --- | --- | --- |
| *N* | *53* | *72* |
| Women | 32 (60.4%) | 40 (55.6%) |
| Age | 73.6 (1.2) | 71.7 (1.3) |
| Education (years) | 12.2 (.6) | 12.7 (.4) |
| GDS (pre-stroke) | 1.2 (.7) | 1.3 (.7) |
| mRS | 2.0 (.1) | 2.1 (.2) |
| Charlson comorbidity (CCI) | 3.5 (.2) | 3.9 (.2) |
| NIHSS | 4.8 (1.0) | 3.3 (.4) |
| MoCA 18 months | 25.5 (.5) | 24.8 (.5) |
| NCD normal | 30 (56.6%) | 38 (52.8%) |
| NCD mild | 17 (32.1%) | 22 (30.6%) |
| NCD major | 6 (11.3%) | 12 (16.7%) |
| WMH (mL) | 14.2 (1.8) | 23.9 (2.9) |
| Stroke size (mL) | 10.5 (3.1) | 6.8 (1.6) |
| Med temp thick (mm) | 2.91 (.02) | 2.88 (.02) |

*Supp. Table 7: NCD from 18 months and mRS from baseline. (N) = Data missing. GDS = Global deterioration scale. mRS = modified Rankin Scale. NIHSS = National Institutes of Health Stroke Scale. MoCA = Montreal Cognitive Assessment. NCD = Neurocognitive disorder. WMH = White matter hyperintensities.*

*Frailty:* ***0-4.*** *Fatigue + stairs + walk + illnesses + weight loss.*

*GDS:* ***1-7****. 2-3 = MCI. 4-7 = Dementia.*

*mRS:* ***0-6.***

*CCI:* ***0-29****.*

*NIHSS:* ***0-42****.*

*MoCA:* ***0-30.***
